# Characteristics of bile acid composition in high fat diet-induced nonalcoholic fatty liver disease in obese diabetic rats

**DOI:** 10.1371/journal.pone.0247303

**Published:** 2021-02-24

**Authors:** Yukiomi Nakade, Rena Kitano, Kazumasa Sakamoto, Satoshi Kimoto, Taeko Yamauchi, Tadahisa Inoue, Yuji Kobayashi, Tomohiko Ohashi, Yoshio Sumida, Kiyoaki Ito, Masashi Yoneda

**Affiliations:** Division of Internal Medicine, Department of Gastroenterology and Hepatology, Aichi Medical University, Nagakute, Aichi, Japan; Max Delbruck Centrum fur Molekulare Medizin Berlin Buch, GERMANY

## Abstract

Bile acid has attracted attention as a signal transmission molecule in energy metabolism. Although a high-fat diet (HFD) or obesity is known to increase hepatic fat content and alter bile acid composition, the changes in bile acid composition due to HFD or obesity remain to be elucidated. We sought to examine the bile acid composition in high fat diet-induced non-alcoholic fatty liver disease (NAFLD) in obese diabetic rats. Eight-week-old male spontaneously diabetic Torii fatty (SDTF) rats or control rats were fed an HFD. Twelve weeks post the commencement of HFD, serum and hepatic bile acid compositions and serum GLP-1 levels, which is stimulated by the secondary bile acid deoxycholic acid (DCA), were measured. The correlation between the bile acid composition and serum GLP-1 levels was also examined. While serum and hepatic levels of cholic acid (CA), a primary bile acid, tended to decrease in HFD-fed control rats, they were significantly decreased in HFD-fed SDTF rats. Hepatic CYP8B1, which plays a role in CA synthesis, the mRNA levels were significantly decreased in HFD-fed control and SDTF rats. In contrast, while serum and hepatic DCA levels were not changed in HFD-fed control rats, they were decreased in HFD-fed SDTF rats. Hepatic DCA/CA did not change in HFD-fed SDTF rats, but significantly increased in HFD-fed control rats. While serum GLP-1 levels were not changed in SDTF rats, they were significantly increased in HFD-fed control rats. Hepatic DCA/CA tended to correlate with serum GLP-1 levels, which tended to negatively correlate with the hepatic triglyceride content in SDTF rats. These results indicate that relatively increased DCA might contribute to an increase in serum GLP-1 levels, which inhibits hepatic steatosis in NAFLD.

## Introduction

With increase in the Westernization of dietary patterns, the prevalence of non-alcoholic fatty liver disease (NAFLD) has risen to 25% in the global population [1]. The incidence of non-alcoholic steatohepatitis (NASH), which lies in the spectrum of NAFLD, has also increased; indeed, NASH is the second leading cause of liver disease among adults awaiting liver transplantation in the United States [2, 3]. Although the pathogenesis of NASH remains unclear, insulin resistance and obesity are considered to play important roles in NASH progression. In recent years, many parallel factors derived from the adipose tissue and gut have been thought to promote liver inflammation [4]. Endoplasmic reticulum stress and its related signaling networks, adipocytokines, and innate immunity are emerging as central factors that regulate key features of NASH [4].

Bile acid has been regarded as the element that plays an important role in fat absorption in the intestine [5]. In recent years, bile acid has attracted attention as a signal transmission molecule in energy metabolism. Bile acid ligates to the farnesoid X receptor (FXR), which is a nuclear receptor expressed in the liver, and attenuates CYP7A1, inhibits the manifestation of Na+-taurocholate co-transporting polypeptide (NTCP), as well as increases bile salt export pump (BSEP) in the liver, resulting in the decrease of bile acid in the liver [6]. On the other hand, deoxycholic acid (DCA), one of the secondary bile acids produced from cholic acid (CA), and DCA also colligates with TGR5, a G protein conjugation receptor, to promote energy metabolism in brown adipose tissues and muscle, resulting in the improvement of overweightness and insulin resistance [7]. TGR5 is also expressed in the small intestine, and DCA ligates to TGR5, which is also manifested in the small intestine and promotes glucagon-like peptide-1(GLP-1) secretion [7].

GLP-1, which is secreted from enteroendocrine L cells, acts on pancreatic β-cells, leading to the promotion of insulin secretion and β-cell proliferation in the pancreas, which results in the control of blood glucose levels [8, 9]. Liraglutide, a novel GLP-1 analog, improves insulin secretion in patients with diabetes [9]. GLP-1 not only regulates blood glucose levels but also induces satiety and regulates gastrointestinal motor functions [10, 11]. With regard to lipid metabolism in the liver, GLP-1 analog decreases high-fat diet-induced hepatic steatosis and inflammation in obese rats and mice [12, 13]. In humans, exenatide, another GLP-1 analog, decreases hepatic fat accumulation [14]. A multicentric double-blinded placebo-controlled trial has shown that liraglutide leads to histological resolution of NASH [15].

Although a high-fat diet or obesity increases hepatic fat content and alters bile acid composition, changes in the bile acid composition due to a high-fat diet or obesity remain to be determined. Furthermore, the relationship between endogenous GLP-1 levels and alterations in bile acid components remains to be elucidated. In the present study, we sought to examine the serum and hepatic levels of bile acids, and the relationship between endogenous GLP-1 levels and changes in bile acid composition in an obese diabetic NAFLD model.

## Materials and methods

### Substances and treatments

High fat diet 32 (HFD) purchased from Japan CLEA Inc. (Tokyo, Japan) was used in the NAFLD model, while AIN-93 purchased from Oriental Yeast Inc. (Tokyo, Japan) was used as the normal diet (control). Both diets were obtained in the powdered form.

### Animal model and experimental design

Eight-week-old male spontaneously diabetic Torii fatty (SDTF) rats, which are known to be obese type II diabetes models, were obtained from Japan CLEA Inc. Eight-week-old male Sprague-Dawley (SD) rats were used as controls. After a 1-week acclimatization period on a control normal diet, 8 SD rats and 8 SDTF rats were fed either a normal diet or HFD for 12 weeks. All rats were given free access to water and experimental diets. Body weights and food consumption in each group of rats were recorded weekly. Protocols describing the use of rats were approved by the Institutional Animal Care and Use Committee of Aichi Medical University (approved number: 2013–53) and were in accordance with the National Institutes of Health "Guide for the Care and Use of Laboratory Animals". After 12 weeks of experimental diet, rats were fasted for 12 h, and blood samples were taken from the retrobulbar, intraorbital, and capillary plexus. Then, 1500 mg/kg D-glucose (British Drug Houses Ltd, Poole, Dorset, UK) under isoflurane anesthesia was administered to the rats through a gavage tube (volume load 0.25 mL), and blood samples were taken at intervals of 30 min, from 30 min to 120 min, after the gavage,. After blood sampling, rats were fed with either a normal diet or HFD for 12 h, and then were fasted for 12 h. The rats were sacrificed using CO_2_ inhalation, and the livers and sub-epididymal fat were rapidly excised, then either fixed in buffered formalin (10%) or frozen in liquid nitrogen and stored at -80°C. Blood samples were collected from the left ventricle, centrifuged, and the serum was stored at -80°C.

### Serum and tissue biochemical measurements

Serum alanine aminotransferase (ALT) and fasting blood glucose (FBG) levels were examined using commercially available kits (Wako, Osaka, Japan). Serum immunoreactive insulin (IRI) levels were measured using an insulin ELISA kit (Funakoshi, Tokyo, Japan), and the homeostasis model assessment-insulin resistance (HOMA-IR) was calculated. Serum GLP-1 levels were also measured using a GLP-1 ELISA kit (Wako, Osaka, Japan). Stored liver samples (100 mg) were lysed and homogenized in 2 mL of a solution containing 150 mM NaCl, 0.1% TritonX-100, and 10 nM Tris using a polytron homogenizer (NS-310E; MicroTech Nichion, Tokyo, Japan) for 1 min. Triglyceride (TG) content in the liver was measured using a Triglyceride Detection Kit (Wako).

### Histopathological examination

Five-micrometer-thick sections from liver tissues fixed in formalin and embedded in paraffin were examined in all experiments. Oil Red O staining was performed using a standard technique to assess hepatic fat deposition. The Oil Red O-positive area was quantified in 5 randomly selected fields per section. The percentage of Oil Red O-positive area was measured using a computerized image analysis system with Image-Pro Plus version 4.5 (Media Cybernetics, Silver Spring, MD).

### Real-time polymerase chain reaction of liver RNA

As described previously [16], frozen liver tissues were homogenized using TRIzol reagent (Life Technologies, Tokyo, Japan), and RNA extraction was performed using an RNeasy Mini Kit (Qiagen, Tokyo, Japan). The isolated RNA was re-suspended in 40 μL of RNase-free water and quantified by spectrophotometry (optical density [OD] 260 and low-mass gel electrophoresis [Invitrogen, Tokyo, Japan]). The total RNA extracted was reverse-transcribed to cDNA using a High Capacity cDNA Reverse Transcription kit (Applied Biosystems, Foster City, CA) according to the manufacturer’s instructions. Real-time quantitative PCR was carried out using ABI Step One Sequence Detection System (Applied Biosystems), and TaqMan PCR was carried out using TaqMan Gene Expression Assays (acyl-coenzyme A oxidase 1 [ACOX1], Rn01460628_m1; [CYP7A1], Rn00564065_m1; [CYP8B1], Rn01445029_s1; [FXR], Rn00572658_m1; microsomal triglyceride transfer protein [MTTP], Rn01522963_m1; sterol regulatory element binding transcription factor 1 [SREBF1], Rn01495769_m1) and TaqMan Universal PCR Master Mix (Applied Biosystems) according to the manufacturer’s instructions. The detailed protocol used for TaqMan PCR was described in a previous study [17].

### Analytical methods for liquid chromatography-mass spectrometry (LC/MS)

The rat livers were homogenized and sonicated sequentially in 80% methanol/water and chloroform/methanol (2:1, v/v). After centrifugation, the supernatant was diluted four times with water and applied to a Bond Elute C18 cartridge (500 mg/6 mL; Varian, Harbor City, CA, USA), which was then washed with 25% ethanol (5 mL), and bile acids were eluted with ethanol (5 mL). After the solvent was evaporated, the residue was dissolved in 1 mL of 50% ethanol. To a 100 μL aliquot of this solution, 0.9 mL of 50% ethanol and 1 mL of IS ([2, 2, 4, 4-d4] CA, 200 pmol/mL in 50% ethanol) was added. Precipitated solids were removed by filtration through a 0.45 μm Millipore filter (Millex^®^-LG; Billerica, MA). A 10 μL aliquot of the filtrate was injected into the LC/MS system, which consisted of a TSQ Quantum Discovery Max mass spectrometer (Thermo Fisher Scientific, San Jose, CA) equipped with an ESI probe and a Surveyor HPLC system (Thermo Fisher Scientific). Aliquots of the plasma (0.1 mL) collected were spiked with internal standard, mixed with 1 mL ice-cold acetonitrile, vortexed, and centrifuged. Supernatant was dried under nitrogen at 45°C and resuspended in 100 mL methanol/water, containing 5 mmol/L ammonium formate. Similarly, a 10-μL aliquot was injected into the LC/MS system. Quantitative standard curves were used, and deuterated internal standards were used to measure recovery. Following LC/MS system, we measured total bile acid (TBA), CA, and DCA levels, and also measured levels of 12αOH BA, which is thought to be associated with insulin resistance and non 12αOH BA levels.

### Statistical analysis

All statistical analyses were performed using BellCurve for Excel version 3.21. All results are expressed as mean ± SE. Comparisons of the values between the two groups were calculated by Student’s t-test. The comparison of serum GLP-1 levels after D-glucose injection with basal levels was conducted by repeated measurements of the analysis of variance (ANOVA). Multiple group comparisons were performed using analysis of variance (ANOVA), followed by Bonferroni’s post hoc test. The correlation coefficient between different variables was determined using the Pearson test, where the values *r* = 1 and −1 represent perfect positive correlation and perfect negative correlation, respectively. A p value < 0.05 was considered statistically significant.

## Results

### Changes in hepatic steatosis, hepatic TG, and FFA contents

**Fig 1:**
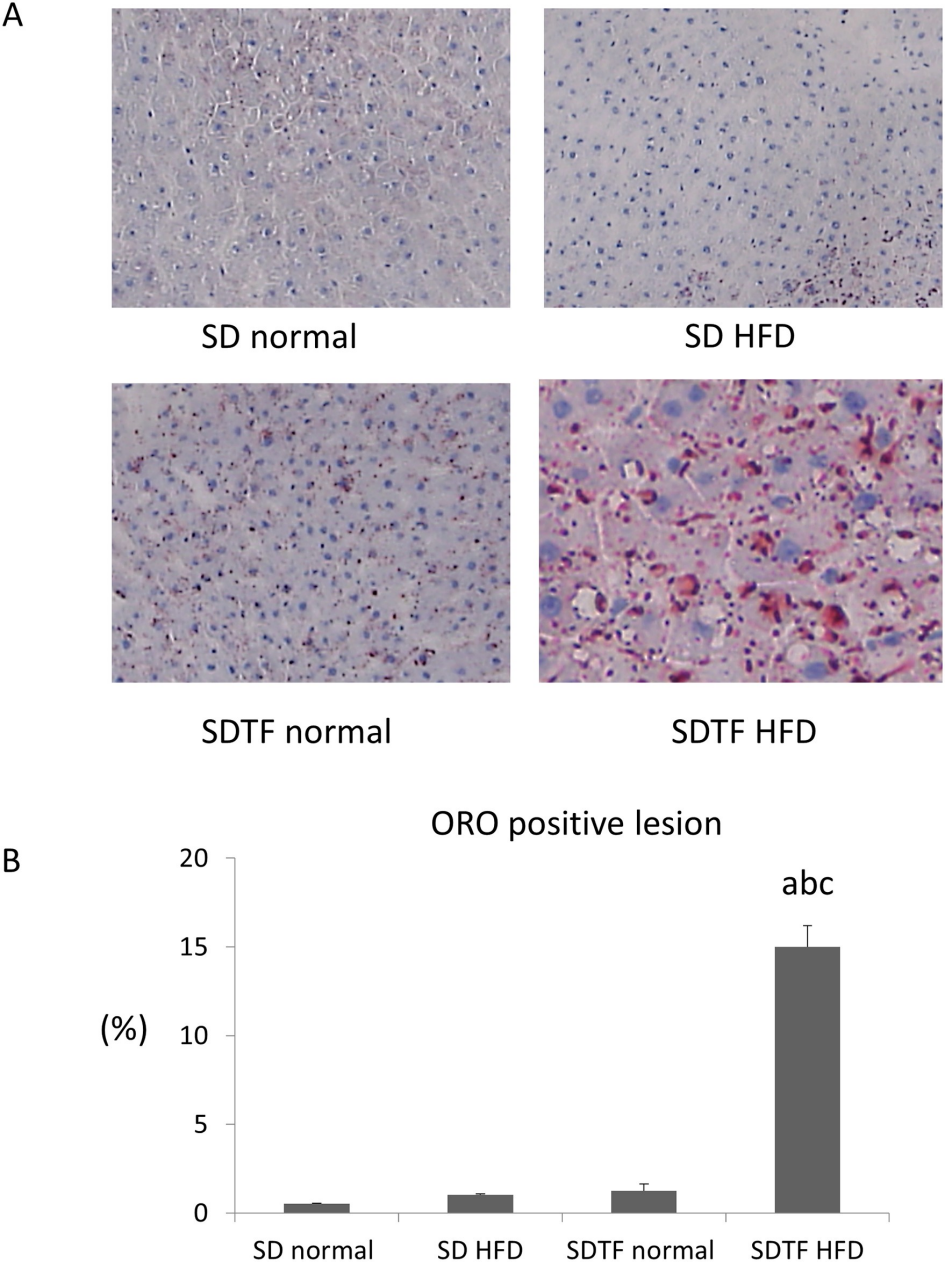
Evaluation of hepatic lipid contents in SD and SDTF rats fed a normal diet and HFD for 12 weeks. (A) Frozen liver sections were stained with Oil Red O. (B) Quantitative analysis of changes in Oil Red O positive area in respective groups. Data are expressed as means ± SE. (^a^ P < 0.01 compared with normal diet-fed SD rats, ^b^ P < 0.01 compared with HFD-fed SD rats, ^c^ P < 0.01 compared with normal diet-fed SDTF rats). Original magnification, 100×.

The body weights of HFD-fed SD and SDTF rats significantly increased in comparison with normal diet-fed SD and SDTF rats, respectively (Table 1). Both the HFD-fed SD and SDTF rats gained sub-epididymal fat weight (Table 1). Oil Red O (ORO) staining showed that HFD significantly increased ORO-positive area in the SDTF rats; however, HFD did not increase ORO-positive area in the SD rats (Fig 1A and 1B). HFD significantly increased hepatic TG contents in the SDTF rats, but not in SD rats (Table 1). HFD did not alter serum ALT levels in either the SD or SDTF rats (Table 1). On the other hand, HFD significantly decreased serum TG levels in both SD and SDTF rats (Table 1).

**Table 1:** Clinical characteristics of rats after 12 weeks of feeding with experimental diets.

Group	n	Body weight gain (g)	Subepididymal fat weight (g)	Hepatic TG(mg/dl)	Serum TG(mg/dl)	Serum ALT (IU/l)
**SD normal**	4	213 ± 26	4.86 ± 0.47	126 ± 8.7	125 ± 7.8	20 ± 3.4
**SD HFD**	4	271 ± 18^a^	8.63 ± 1.05^a^	157 ± 6.4	69 ± 4^a^	23 ± 3.2
**SDTF normal**	4	127 ± 16 ^a^	7.47 ± 0.64 ^a^	113 ± 17	432 ± 68 ^a^	44 ± 3.4
**SDTF HFD**	4	263 ± 23^a^^b^	14.9 ± 0.53^a^^b^	244 ± 13^a^^b^	251 ± 25^a^^b^	49 ± 8.5

Data are expressed as means ± SE; Statistical comparison were made using one-way ANOVA.

^a^; significantly different from normal diet-fed SD rats (P < 0.05).

^b^; significantly different from normal diet-fed SDTF rats (P < 0.05).

### Changes in fasting blood glucose, serum IRI, and HOMA-IR levels

Fasting blood glucose (FBG) levels were significantly higher in normal diet-fed SDTF rats than in the control diet-fed SD rats (Table 2). HFD significantly attenuated FBG levels in SDTF rats (Table 2). HFD tended to increase serum IRI levels in SD and SDTF rats (Table 2). While serum IRI levels were significantly attenuated in the normal diet-fed SDTF rats compared with normal diet-fed SD rats, serum IRI levels tended to be higher in HFD-fed SDTF rats (Table 2). On the other hand, HOMA-IR levels tended to decrease in the HFD-fed SD and SDTF rats (Table 2).

**Table 2:** Clinical characteristics of rats fed with experimental diets.

Group	n	FBG (mg/dL)	IRI(μg/L)	HOMA-IR
**SD normal**	4	166 ± 16	5.77 ± 1.36	2.29 ± 1.36
**SD HFD**	4	121 ± 25	6.39 ± 0.74	1.78 ± 0.15
**SDTF normal**	4	519 ± 44 ^a^	2.14 ± 0.43 ^a^	2.75 ± 0.62
**SDTF HFD**	4	387 ± 29 ^a^^b^	2.51 ± 0.11 ^a^	1.91 ± 0.10

Data are expressed as means ± SE; Statistical comparison were made using one-way ANOVA.

^a^; significantly different from normal diet-fed SD rats (P < 0.05).

^b^; significantly different from normal diet-fed SDTF rats (P < 0.05).

### Changes in serum GLP-1 levels in SD and SDTF rats

**Fig 2:**
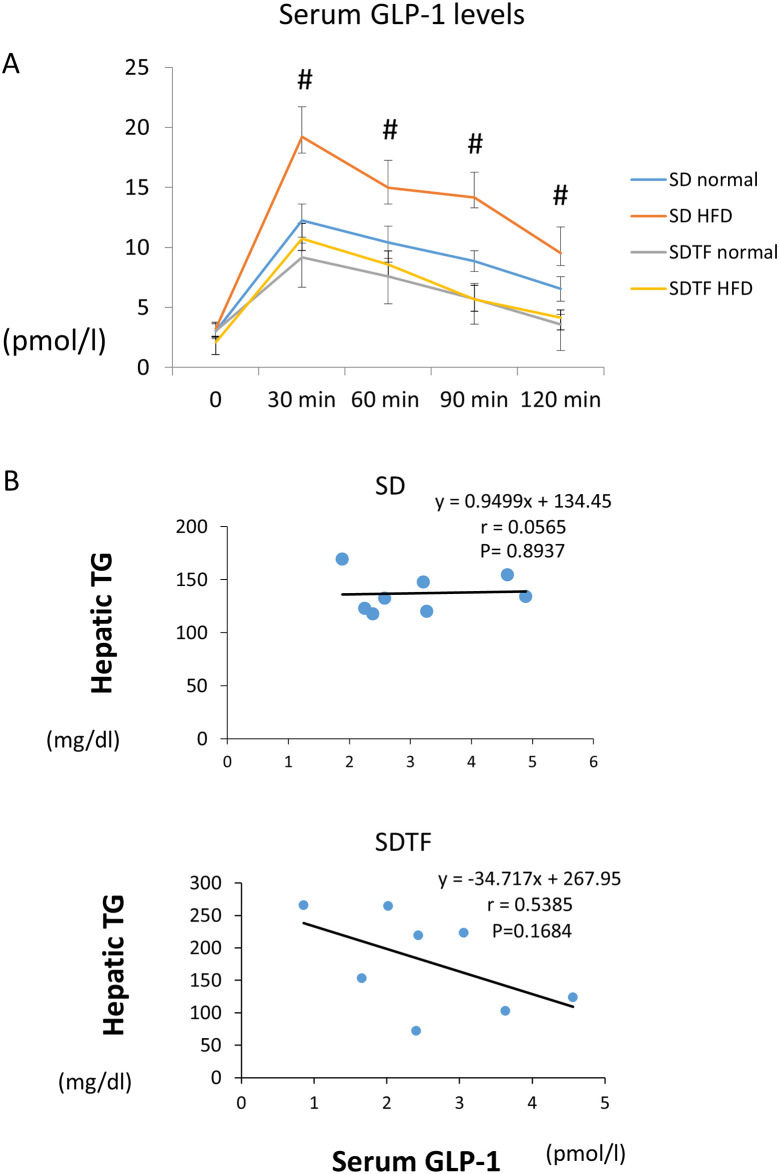
Evaluation of serum GLP-1 levels and the correlation between GLP-1 levels and hepatic TG contents. (**A**) Time course changes for serum GLP-1 levels following intragastric D-glucose in SD and SDTF rats fed normal diet and HFD. (**B**) The correlation between hepatic triglyceride contents and serum GLP-1 levels in SD and SDTF rat groups. Data are expressed as means ± SE. (^#^ P < 0.05 compared with normal diet-fed SD rats).

Fasting GLP-1 levels did not change among the four groups in both the normal diet- and HFD-fed SD and SDTF rats (Fig 2A). Following intragastric D-glucose, GLP-1 levels in plasma quickly reached a peak level at 30 min and gradually decreased over the next 90 min in both normal diet and HFD-fed SD and SDTF rats (Fig 2A). The peak concentration of GLP-1 in the HFD-fed SD rats was significantly higher than that in the control diet-fed SD rats (Fig 2A). In contrast, the peak concentration of GLP-1 in the HFD-fed SDTF rats was not changed compared with that in the normal diet-fed SDTF rats (Fig 2A). Hepatic TG contents tended to be negatively correlated with the peak concentration of GLP-1 in the SDTF rat groups (Fig 2B). On the other hand, hepatic TG contents were not correlated with the peak concentration of GLP-1 in SD rat groups (Fig 2B).

### Changes in serum and hepatic bile acid levels in SD and SDTF rats

**Fig 3:**
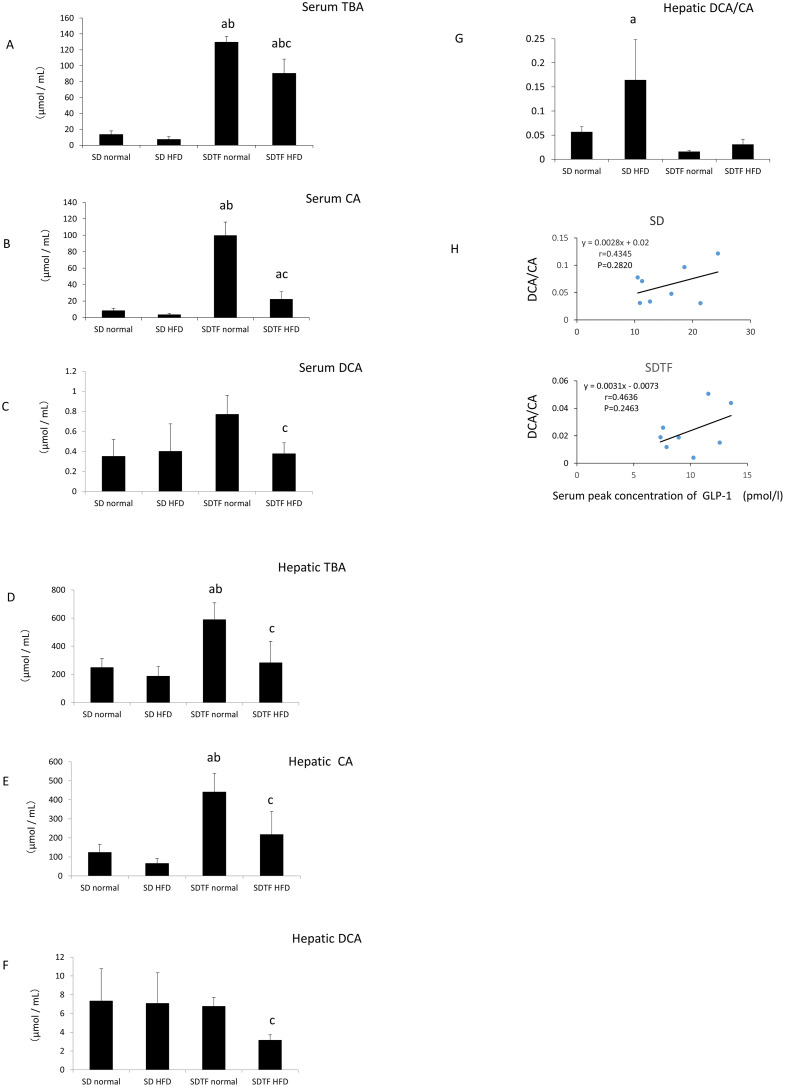
Evaluation of serum or hepatic TBA, CA, and DCA levels. Serum TBA (A), CA (B), and DCA (C) levels and hepatic TBA (D), CA (E), and DCA (F) levels in the SD and SDTF rats fed normal diets and HFD. Hepatic DCA/CA in SD and SDTF rats (G). The correlation between hepatic DCA/CA and the peak concentration of GLP-1 in the serum in both SD and SDTF rat groups (H). Data are expressed as means ± SE. (^a^ P < 0.05 compared with normal diet-fed SD rats, ^b^ P < 0.05 compared with HFD-fed SD rats, ^c^ P < 0.05 compared with normal diet-fed SDTF rats).

With regard to the composition of bile acid in both serum and liver tissues, TBA and CA levels tended to decrease in the HFD-fed SD rats (Fig 3A, 3B, 3D and 3E). In contrast, serum and hepatic DCA levels were not changed in the HFD-fed SD rats (Fig 3C and 3F). Both serum and hepatic TBA and CA levels were significantly augmented in the normal diet-fed SDTF rats compared with that in the normal diet-fed SD rats (Fig 3A, 3B, 3D and 3E). On the other hand, serum and hepatic DCA levels were not augmented in the normal diet-fed SDTF rats (Fig 3C and 3F). HFD significantly decreased both serum and hepatic TBA, CA, and DCA levels in SDTF rats (Fig 3A–3F). Hepatic DCA/CA was significantly increased in the HFD-fed SD rats (Fig 3G), while it was unchanged in HFD-fed SDTF rats (Fig 3G). Hepatic DCA/CA tended to positively correlate with the peak concentration of GLP-1 in the serum in both SD and SDTF rat groups, respectively (Fig 3H).

**Fig 4:**
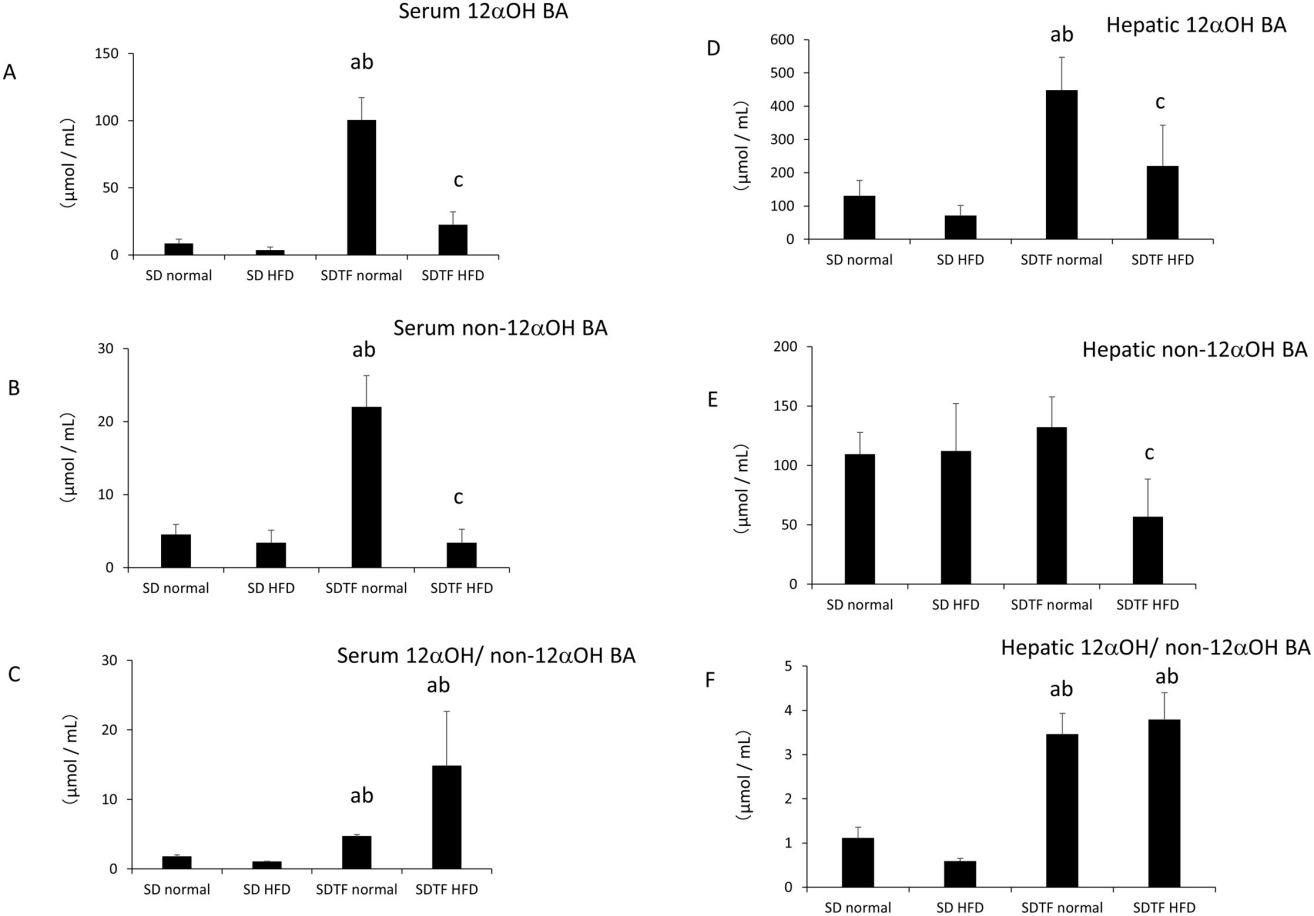
Evaluation of serum or hepatic 12αOH BA and non-12αOH BA levels. Serum 12αOH BA levels (A), serum non-12αOH BA levels (B), serum 12αOH BA/non-12αOH BA (C) and hepatic 12αOH BA levels (D), hepatic non-12αOH BA levels (E), hepatic 12αOH BA/non-12αOH BA (F) in SD and SDTF rats-fed normal diets and HFD. Data are expressed as means ± SE. (^a^ P < 0.05 compared with normal diet-fed SD rats, ^b^ P < 0.05 compared with HFD-fed SD rats, ^c^ P < 0.05 compared with normal diet-fed SDTF rats).

Both serum and hepatic 12αOH BA levels were not changed by HFD in SD rats (Fig 4A and 4D). In contrast, both serum and hepatic 12αOH BA levels were significantly augmented in control diet-fed SDTF rats compared with that in control diet-fed SD rats (Fig 4A and 4D). HFD significantly decreased both serum and hepatic 12αOH BA levels in the SDTF rats (Fig 4A and 4D). While serum non-12αOH BA levels were significantly increased, hepatic non-12αOH BA levels were not changed in control diet-fed SDTF rats (Fig 4B and 4E). Serum and hepatic non-12αOH BA levels were significantly decreased in the HFD-fed SDTF rats (Fig 4B and 4E). Both serum and hepatic 12αOH/non-12αOH BA were increased in the normal diet-fed SDTF rats (Fig 4C and 4F). HFD did not change 12αOH/non-12αOH BA levels in HFD-fed SDTF rats (Fig 4C and 4F).

### Changes in hepatic bile acid-related gene expression

**Fig 5:**
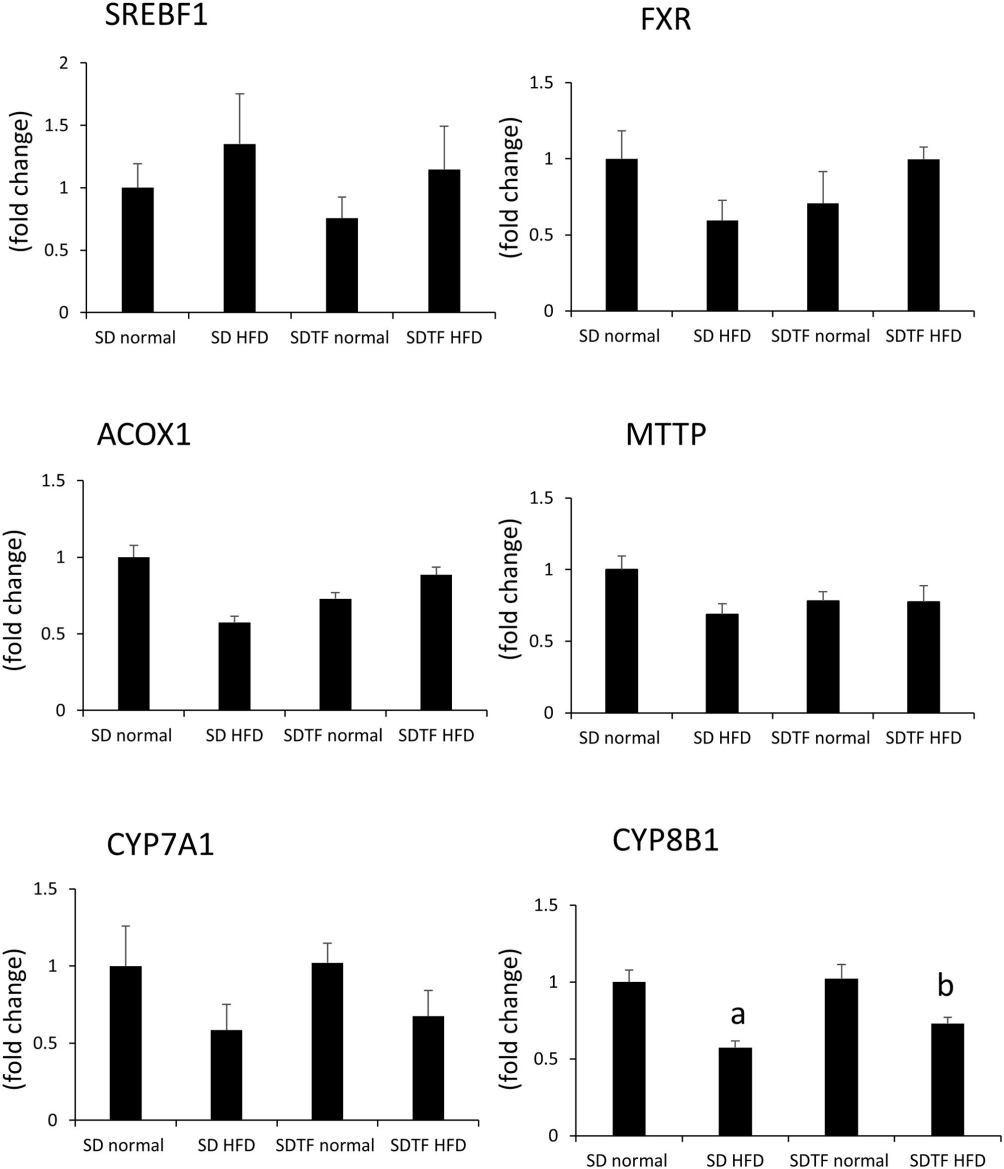
Evaluation of hepatic bile acid metabolism-related genes in SD and SDTF rats fed normal diet and HFD. Each chart details the *SREBF1*, *FXR*, *ACOX1*, *MTTP*, *CYP7A1*, and *CYP8B1* mRNA levels. Data are expressed as means ± SE. (^a^ P < 0.05 compared with normal diet-fed SD rats, ^b^ P < 0.05 compared with normal diet-fed SDTF rats).

Hepatic SREBF1 levels are related to *de novo* lipogenesis, and mRNA levels tended to increase in HFD-fed SD and SDTF rats (Fig 5). Hepatic FXR is a member of the nuclear receptor superfamily and is activated by bile acids. The mRNA levels of *FXR* did not significantly change in either the control diet or HFD-fed SD and SDTF rats (Fig 5). The mRNA levels of *ACOX1* and *MTTP*, which are associated with hepatic TG excretion, did not significantly change in either control diet or HFD-fed SD and SDTF rats (Fig 5). In case of hepatic CYP7A1, which is the rate-limiting enzyme in bile acid synthesis, the mRNA levels were unchanged in both control and HFD-fed SD rats (Fig 5). In contrast, in the case of hepatic CYP8B, which plays a role in CA synthesis, the mRNA levels were significantly decreased in HFD-fed SD and SDTF rats (Fig 5).

## Discussion

With regard to the correlation between hepatic steatosis and bile acid, Aranha et al. reported that bile acid levels in the liver, as well as hepatic DCA, CDCA, and CA levels were elevated in patients with steatohepatitis [18]. Furthermore, significant correlations were found between hepatic CA levels and fibrosis in patients with NASH [18]. Lake et al. reported that the BA metabolomic profile of NASH livers exhibited increased levels of taurine, conjugated BA species, taurocholic acid, and taurodeoxycholic acid, but decreased levels of CA and glycodeoxycholic acid [19]. Another study reported that hydroxycholic acid was the main alteration in hepatic bile acid composition in a hypertensive NAFLD model [20]. Thus, there is no confirmed relationship between hepatic steatosis and bile acid levels.

In the present study, serum and hepatic TBA levels significantly increased in normal diet-fed SDTF rats in comparison with normal diet-fed SD rats. We observed an increase in not only serum and hepatic CA levels, but also DCA levels in normal diet-fed SDTF rats. These results indicated that genetic obesity and diabetes may affect serum and hepatic bile acid levels. The reason for these trends remains to be determined. The body weight gain in normal diet-fed SDTF rats was lower than that in normal diet-fed SD rats. Serum ALT and hepatic TG levels were not significantly increased in normal diet-fed SDTF rats. The significantly higher glucose levels in normal diet-fed SDTF rats compared with those in normal diet-fed SD rats might contribute to the impairment in body weight gain. Previous reports indicated that bile acid levels were increased in rats that underwent bariatric surgery [21]. The decrease in body weight or impairment of body weight gain might contribute to the increase in TBA levels.

HFD produced hepatic steatosis in SDTF rats, but not in SD rats. While serum GLP-1 levels were not significantly increased in HFD-fed SDTF rats post D-glucose administration, they were significantly increased in HFD-fed SD rats. Liver steatosis caused by hepatic TG contents tended to negatively correlate with serum GLP-1 levels in SDTF rats. Although serum and hepatic bile acid levels did not increase, serum GLP-1 levels were augmented post D-glucose administration in SD rats. While HFD tended to attenuate serum and hepatic TBA and CA levels in SD rats, HFD significantly decreased serum and hepatic TBA and CA levels in SDTF rats. Serum and hepatic DCA levels were not changed in the HFD-fed SD rats, but were attenuated in HFD-fed SDTF rats. While hepatic DCA/CA was significantly increased in HFD-fed SD rats, it was unchanged in HFD-fed SDTF rats. Hepatic DCA/CA tended to be positively correlated with serum GLP-1 levels. DCA is produced from CA by enterobacteria [22]. Increased DCA/CA ratio seen in HFD-fed SD rats might be caused by changes in the gut microbiota. A high DCA/CA ratio has been observed in patients with excess DCA [23]. DCA has been reported to bind to TGR5 in L cells from the mucosal side, resulting in an increase in serum GLP-1 levels [7]. These results indicate that relatively increased DCA levels might contribute to an increase in serum GLP-1 levels in SD rats.

On the other hand, not only GLP-1, but also fibroblast growth factor 21, which is a negative regulator of bile acid synthesis, has a beneficial effect for NASH patients [24]. A recent phase 2 trial of FGF 21 analogue improved metabolic parameters and fibrosis biomarker [25]. It is of interest to examine the association between bile acid and FGF 21 levels in this model.

Although fatty liver was induced in HFD-fed SDTF rats, serum TG levels were not increased. The reason for this observation in HFD-fed SDTF rats remains to be elucidated. Since previous reports have shown that VLDL-TG excretion is impaired in NAFLD patients [26], hepatic VLDL-TG excretion might be impaired in this model. However, the reason why serum ALT levels were not increased in HFD-fed SDTF rats remains to be determined. Previous reports have reported the induction of fatty liver in female normal diet-fed SDTF rats; however, such induction is unclear in male normal diet-fed SDTF rats [27]. With regard to the sex-related differences in the effects of NAFLD, NAFLD occurs at a higher rate in women after menopause, suggesting that estrogen has a protective effect [28]. Estrogen levels might be involved in hepatic steatosis of normal diet-fed SDTF rats.

With regard to the correlation between insulin resistance and bile acid, human insulin resistance is associated with increased serum levels of 12αOH BA [29]. We showed that HOMA-IR levels tended to increase, serum and hepatic 12αOH BA levels significantly augmented in control diet-fed SDTF rats compared with those in control diet-fed SD rats. In contrast, HFD tended to decrease HOMA-IR levels, and significantly decreased serum and hepatic 12αOH BA levels in SDTF rats. We also showed that serum 12αOH/non-12αOH BA levels were significantly increased in SDTF rats. Previous reports indicated that high 12αOH/non-12αOH BA were associated with key features of insulin resistance [29]. HOMA-IR tended to increase in SDTF rats. These results indicate that HFD alone may not affect insulin resistance; however, genetic obese and diabetic factor in SDTF rats might affect serum and hepatic 12αOH/non-12αOH BA, resulting in increased insulin resistance.

We investigated hepatic bile acid metabolism-related gene expression mediated by HFD in SD and SDTF rats. We showed that hepatic *SREBF1* mRNA levels tended to increase in HFD-fed SD and SDTF rats, indicating that HFD may induce *de novo* lipogenesis. On the other hand, hepatic *FXR*, *ACOX1*, *MTTP* mRNA levels did not significantly change in either normal diet or HFD-fed SD and SDTF rats; this indicates that hepatic bile acid composition alteration may not affect hepatic β-oxidation, hepatic TG excretion. In contrast, hepatic *CYP8B1*mRNA levels were significantly decreased in HFD-fed SDTF rats. A previous report indicated that insulin inhibited the production of 12αOH BA by suppressing the transcription of *CYP8B1* [30]. We showed that serum IRI levels tended to be increased, and hepatic 12αOH BA levels tended to be or was significantly decreased in HFD-fed SD or SDTF rats. These results indicate that HFD-induced serum insulin levels increase may contribute to the reduction of *CYP8B1* mRNA levels. In the bile acid synthesis pathway, CA is synthesized by *CYP8B1* [31]. On the other hand, HFD decreased hepatic CA levels in SD and SDTF rats. These results indicated that HFD induced augmentation of insulin may suppress the transcription of *CYP8B1* resulting in the inhibition of CA synthesis.

In conclusion, while the hepatic DCA/CA did not change in HFD-fed SDTF rats, it significantly increased in HFD-fed SD rats. Hepatic DCA/CA tended to be positively correlated with the peak concentration of GLP-1 in the serum, which tended to be negatively correlated with hepatic TG content. These results indicate that relatively increased DCA might contribute to an increase in serum GLP-1 which inhibits hepatic steatosis.

## Supporting information

S1 Data Data set of figures. (XLSX)

S1 Checklist The ARRIVE guidelines 2.0: Author checklist. (PDF)
